# Relação entre Velocidade de Onda de Pulso e Biomarcadores Cardiovasculares em Pacientes com Fatores de Risco

**DOI:** 10.36660/abc.20190348

**Published:** 2020-12-01

**Authors:** Rayne Ramos Fagundes, Priscila Valverde Oliveira Vitorino, Ellen de Souza Lelis, Paulo Cesar B. Veiga Jardim, Ana Luiza Lima Souza, Thiago de Souza Veiga Jardim, Pedro Miguel Guimarães Marques Cunha, Weimar Kunz Sebba Barroso

**Affiliations:** 1 Universidade Federal de Goiás GoiâniaGO Brasil Universidade Federal de Goiás,Goiânia, GO – Brasil; 2 Pontifícia Universidade Católica de Goiás Escola de Ciências Sociais e da Saúde GoiâniaGO Brasil Pontifícia Universidade Católica de Goiás - Escola de Ciências Sociais e da Saúde, Goiânia, GO - Brasil; 3 Universidade Federal de Goiás Liga de Hipertensão GoiâniaGO Brasil Universidade Federal de Goiás - Liga de Hipertensão, Goiânia, GO - Brasil; 4 Universidade do Minho Escola de Medicina Braga Portugal Universidade do Minho Escola de Medicina, Braga – Portugal

**Keywords:** Doenças Cardiovasculares/mortalidade, Pressão Arterial, Fatores de Risco, Hipertensão, Disfunção Ventricular Esquerda, Diabetes Mellitus

## Abstract

**Fundamento:**

A relação entre velocidade de onda de pulso (VOP) e biomarcadores de mudanças estruturais do ventrículo esquerdo e artérias carótidas ainda é pouco elucidada.

**Objetivo:**

Investigar a relação entre VOP e esses biomarcadores.

**Métodos:**

Estudo transversal, retrospectivo e analítico. Revisamos prontuários médicos de pacientes com diabetes mellitus, dislipidemia, e pré-hipertensão ou hipertensão, que realizaram medida de pressão arterial central (PAC) utilizando o Mobil-O-Graph®, e doppler de carótida ou ecocardiografia três meses antes ou após a medida da PAC. Análise estatística realizada por correlação de Pearson ou de Spearman, análise de regressão múltipla e de regressão bivariada, e teste t (independente) ou de Mann-Whitney. Um p<0,05 indicou significância estatística.

**Resultados:**

Prontuários de 355 pacientes foram avaliados, 56,1 ±14,8 anos, 51% homens. A VOP correlacionou-se com espessuras da íntima média (EIM) das carótidas (r=0,310) do septo do ventrículo esquerdo (r=0,191) e da parede posterior do ventrículo esquerdo (r=0.215), e com diâmetro do átrio esquerdo (r=0,181). A EIM associou-se com VOP ajustada por idade e pressão sistólica periférica (p=0,0004); uma EIM maior que 1mm aumentou em 3,94 vezes a chance de se apresentar VOP acima de 10m/s. A VOP foi significativamente maior em indivíduos com hipertrofia do ventrículo esquerdo (p=0,0001), EIM > 1 mm (p=0,006), placa de carótida (p=0,0001), estenose ≥ 50% (p=0,003), e lesões de órgãos-alvo (p=0,0001).

**Conclusões:**

A VOP correlacionou-se com a EIM e com parâmetros ecocardiográficos, e se associou independentemente com EIM. Essa associação foi mais forte em pacientes com hipertrofia do ventrículo esquerdo, EIM aumentada, placa de carótida, estenose ≥ 50%, e lesões de órgãos-alvo. (Arq Bras Cardiol. 2020; 115(6):1125-1132)

## Introdução

A alta prevalência e a elevada mortalidade das doenças cardiovasculares (DCVs) destacam a urgente necessidade de se implementar ferramentas para melhor estratificação de risco cardiovascular, identificar os pacientes em alto risco, e diagnosticar e tratar precocemente as doenças. Uma dessas ferramentas são os biomarcadores cardiovasculares, os quais conseguem detectar as DCVs em sua fase subclínica, com boa acurácia, melhorando, assim, a prevenção de eventos e o cenário epidemiológico.^1 , 2^

Alguns dos principais biomarcadores relacionados à estrutura e à função vascular são a espessura da íntima-média (EIM), presença de placas na artéria carótida, velocidade de onda de pulso (VOP), e o índice tornozelo-braquial (ITB).^2^Além disso, outros biomarcadores cardiovasculares são usados para identificar lesões de órgãos-alvo (LOA), tais como hipertrofia do ventrículo esquerdo, níveis elevados de creatinina sérica, excreção aumentada de albumina, e taxa de filtração glomerular reduzida.^3 , 4^

A VOP, um marcador de dano vascular utilizado na avaliação de rigidez arterial, é considerada um forte marcador independente de LOA e eventos adversos.^5^ A VOP também é um preditor de mortalidade por todas as causas, indicando o risco real do paciente.^6^ O aumento de um metro por segundo (1m/s) na VOP leva a um aumento de 14% no risco de eventos adversos e de 15% no risco cardiovascular e mortalidade por todas as causas.^6^ Entre suas vantagens, a VOP é um método não invasivo, fácil, de custo relativamente baixo, e amplamente validado,^2^ com valores de referência claramente estabelecidos.^7 , 8^Apesar dessas evidências, a VOP continua subutilizada na prática clínica, e poucos estudos analisaram sua relação com outros biomarcadores, especialmente utilizando o método oscilométrico. Assim, o objetivo deste estudo foi investigar a relação entre a VOP e outros biomarcadores das alterações estruturais cardiovasculares em pacientes com fatores de risco cardiovascular.

## Métodos

### Participantes

De setembro de 2012 a março de 2017, foram realizadas 660 medidas da pressão arterial central (PAC). Entre essas avaliações, 131 pacientes realizaram o exame duas vezes ou mais, totalizando 169 avaliações repetidas. Assim, a população do estudo foi composta por 491 pacientes que realizaram avaliações da PAC.

**Figura 1:**
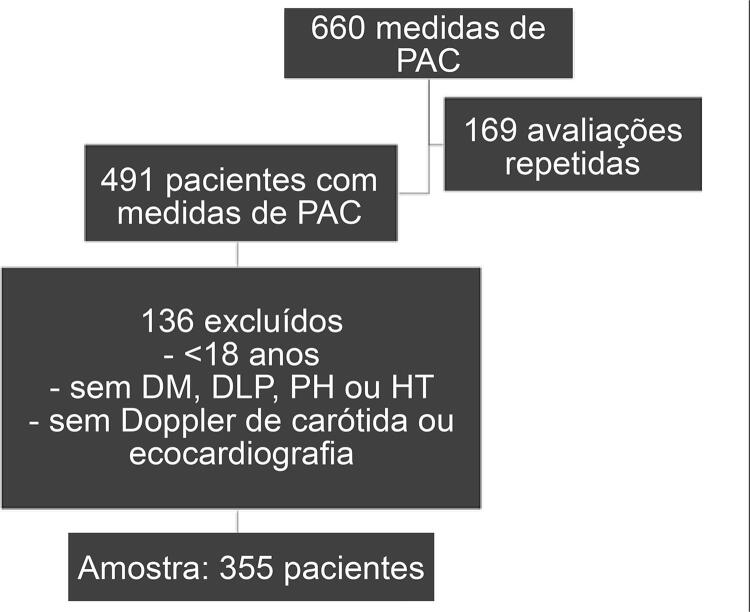
– Fluxograma da seleção da amostra do estudo; PAC: pressão arterial central; DM: diabetes mellitus; PH: pré-hipertensão; HT: hipertensão.

O cálculo da amostra baseou-se em um erro de 5% e nível de confiança de 95%, que indicou um tamanho amostral mínimo de 216 pacientes. A amostra do estudo consistiu em 355 pacientes brasileiros encaminhados a uma clínica de cardiologia para avaliação da PAC ( Figura 1 ).

### Delineamento do Estudo e Procedimentos

Este estudo analítico, retrospectivo, transversal foi realizado a partir da análise de prontuários médicos e laudos de exames. Dados foram primeiramente coletados dos prontuários médicos do serviço de arquivo médico da instituição. Foram aplicados os seguintes critérios de exclusão: idade inferior a 18 anos, ausência dos seguintes diagnósticos: diabetes mellitus (DM), dislipidemia (DLP), pré-hipertensão (PH) ou hipertensão (HT); ausência de Doppler de carótida ou de ecocardiografia nos três meses antecedentes ou posteriores à medida de PAC ( Figura 1 ).

Os diagnósticos de todos os pacientes foram obtidos dos prontuários médicos e, quando esses não estavam disponíveis, os seguintes critérios diagnósticos foram usados – glicemia de jejum > 125 mg/dL ou uso de hipoglicemiantes para DM, níveis de triglicerídeos > 150 mg/dL e de lipoproteína de baixa densidade (LDL) > 100mg/dL e/ou de lipoproteína de alta densidade (HDL) < 40 mg/dL e/ou uso atual de estatinas para dislipidemia. Indivíduos com pressão arterial sistólica (PAS) periférica entre 121 e 139 mmHg e pressão arterial diastólica (PAD) entre 81 e 89 mmHg, medidas durante a avaliação da PAC, foram classificados como pré-hipertensos, e aqueles com pressão arterial igual ou superior a 140/90 mmHg foram classificados como hipertensos.^4^

Dados sobre as seguintes variáveis foram coletadas dos prontuários médicos: sexo (feminino ou masculino), tabagismo (sim ou não), e estado civil (com ou sem parceiro/a). Além dos resultados dos exames de imagem, resultados do Doppler de carótida e/ou ecocardiografia conduzidos nos três meses antes e após o exame de PAC foram analisados. Quando esses exames eram realizados mais de uma vez nesse período, os resultados do último teste antes da medida da PAC foram considerados para análise.

### Medida da PAC

A PAC foi determinada pelo método validado, não-invasivo, oscilométrico, pelo equipamento Mobil-O-Graph^®^ (IEM, Stolberg, Alemanha), com algoritmo ARCSolver.^10^ Todas as medidas foram realizadas pelo mesmo indivíduo, sempre das 13 horas às 14 horas, utilizando a análise tripla de onda de pulso e calibração MAD-c2 (pressão diastólica média).^9 , 10^ Idade foi calculada como a diferença entre a data de nascimento e a data da medida da PAC. Peso (Kg) e altura (m) foram usados para o cálculo do índice de massa corporal (IMC, Kg/m^2^) pela fórmula de Quetelet^11^ e sua classificação.^12^ PAS periférica (PASp), PAD periférica (PADp), PAS central (PASc), índice de aumento (IA), e VOP foram analisados.^13^ Todos os pacientes foram orientados a não fumar ou beber café antes do teste.

### Doppler de Carótida e Ecocardiograma

Os exames de imagem foram realizados em diferentes centros de imagem, segundo escolha do paciente. Exames realizados na clínica de cardiologia em que ocorreu a coleta de dados foram conduzidos com equipamento de ultrassom Philips HD 11.

O Doppler de carótida foi realizado seguindo-se as diretrizes norte-americanas^14^ e europeia.^15^ Os valores mais altos obtidos das artérias carótidas comuns direita e esquerda foram considerados para a análise estatística.

Os parâmetros ecocardiográficos foram avaliados por ecocardiografia transtorácica bidimensional,^16^ com medidas da espessura do septo do ventrículo esquerdo (ESVE), da espessura da parede posterior do ventrículo esquerdo (EPPVE), e do diâmetro do átrio esquerdo (DAE).

### Lesões de Órgãos-alvo

**Figura 2:**
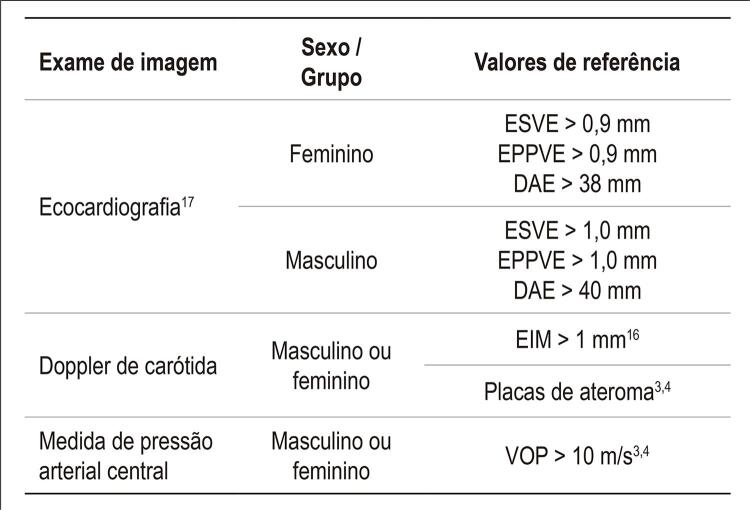
– Exames e valores de referência considerados indicativos de lesões de órgãos-alvo. DAE: diâmetro do átrio esquerdo; EIM: espessura da íntima média; EPPVE: espessura da parede posterior do ventrículo esquerdo; ESVE: espessura do septo do ventrículo esquerdo; VOP: velocidade de onda de pulso.

A identificação de LOA baseou-se na presença de EIM aumentada,^17^ placas de ateroma no Doppler de carótida,^3 , 4^ hipertrofia do ventrículo esquerdo (HVE) no ecocardiograma,^18^ e aumento da rigidez arterial identificado por uma VOP maior que 10m/s^3 , 4^ ( Figura 2 ).

### Análise Estatística

Os dados foram coletados e escaneados em duplicata por dois pesquisadores, utilizando o programa Epidata, versão 3.1. Após analisar e corrigir as inconsistências, os dados foram exportados para o programa Statistical Package for Social Science (SPSS), versão 18.0. O teste de Kolmogorov-Smirnov foi aplicado, e se realizou uma análise descritiva dos dados. A análise estatística foi realizada com base na distribuição dos dados, utilizando testes paramétricos e não paramétricos. Os dados numéricos foram descritos como média e desvio padrão ou média e intervalo interquartil, dependendo da distribuição dos dados. As variáveis categóricas foram apresentadas em frequência absoluta e relativa. O coeficiente de correlação de Pearson ou a correlação de Spearman foram usados para avaliar a correlação da VOP com os resultados obtidos no Doppler de carótida e ecocardiograma. As correlações foram classificadas como fraca (0 < r <0,30), moderada (0,30 ≤ r < 0,60), forte (0,60 ≤ r < 0,90) e muito forte (0,90 ≤ r < 1).^19^

A associação entre a VOP e outros biomarcadores (EIM, ESVE, EPPVE e DAE) foi avaliada por análise de regressão linear bivariada. As variáveis com p <0.020 foram usadas na análise de regressão múltipla. Todas as premissas para a aplicação da análise de regressão linear foram atendidas. A VOP foi comparada segundo magnitude da EIM, presença ou não de HVE, presença ou não de placa, dimensão da placa, e presença ou não de LOA, utilizando-se o teste para amostras independentes ou o teste de Mann-Whitney. Valores de p<0,05 foram considerados estatisticamente significativos.

### Aspectos Éticos

O estudo foi conduzido de acordo com a resolução 466/12 do Conselho Nacional de Saúde, e foi aprovado pelo comitê de ética do Hospital das Clínicas da Universidade Federal de Goiás (UFG) (protocolo de aprovação número 1.500.463).

## Resultados

Um total de 355 indivíduos, com idade média de 56,1 (±14,8) anos participaram no estudo. A maioria apresentou dislipidemia e/ou hipertensão arterial, 148 (41;7%) apresentavam sobrepeso e 130 (36.6%) eram obesos ( Tabela 1 ).

**Tabela 1:** – Características da amostra

Variáveis		Média (DP) / Mediana (25%-75%) / n (%)
Idade		56,1 (±14,8)
IMC		28,7 (±4,9)
PASc		113 (107-123)
IA		21,5 (±13,4)
VOP		8,2 (±2)
Sexo		
Masculino		181 (51%)
Feminino		174 (49%)
**Estado civil**		
Com parceiro		251 (70,7%)
Sem parceiro		102 (28,7%)
**Fatores de risco cardiovasculares**		
Sobrepeso		148 (41.7%)
Obesidade		130 (36,6%)
Tabagismo		12 (3,4%)
**Diagnóstico **		
Dislipidemia		306 (86,2%)
Hipertensão arterial		283 (79,7%)
Diabetes mellitus		65 (18.3%)
Pré-hipertensão		47 (13,2%)

*IA: índice de aumento; IMC: índice de massa corporal; VOP: velocidade de onda de pulso; PASc: pressão arterial sistólica central.*

Uma correlação moderada e positiva foi encontrada entre a VOP e a EIM; e correlações positivas fracas foram identificadas entre a VOP e a ESVE, e entre a EPPVE e DAE) ( Tabela 2 ).

**Tabela 2:** – Correlação da velocidade de onda de pulso e biomarcadores cardiovasculares

		EIM (n=178)	ESVE (n=313)	EPPVE (n=312)	DAE (n=312)
VOP	r	0,310^†^	0,191^†^	0,215^†^	0,181^‡^
	p	<0,001*	0,001*	<0,001*	0,001*

*p < 0.05. †Correlação de Spearman; ‡Correlação de Pearson; EIM: espessura da íntima média; DAE: diâmetro do átrio esquerdo; EPPVE: espessura da parede posterior do ventrículo esquerdo; ESVE: espessura do septo ventricular esquerdo; VOP: velocidade de onda de pulso.

A EIM foi associada com a VOP ajustada para idade e pressão sistólica periférica (p=0.0004). Um aumento de 1mm ou mais na EIM aumentou em 3.94 vezes a chance de se apresentar VOP acima de 10m/s ( Tabelas 3 e 4 ).

**Tabela 3:** – Análise de regressão linear bivariada da velocidade de onda de pulso com biomarcadores cardiovasculares

Variáveis	OR	IC95% (OR)	p
ESVE	2,49	1,38 – 4,49	0,003*
EIM	3,94	1,53 – 10,15	0,004*
EPPVE	2,34	1,29 – 4,22	0,005*
DAE	2,55	1,18 – 5,49	0,017*

IC: intervalo de confiança; EIM: espessura da íntima média; DAE: diâmetro do átrio esquerdo; EPPVE: espessura da parede posterior do ventrículo esquerdo; ESVE: espessura do septo ventricular esquerdo; VOP: velocidade de onda de pulso; OR: odds ratio; * p < 0,05.

**Tabela 4:** – Análise de regressão múltipla da velocidade de onda de pulso com biomarcadores cardiovasculares

Variáveis	OR ajustado	IC 95% (OR)	p	OR ajustado*	IC 95% (OR)	p
LVST	1,64	0,59-4,5	0,340	-	-	-
IMT	3,94	1,53- 10,15	0,004	6,86	1,78-26,45	<0,001
LVPWT	1,69	0,64-4,49	0,294	-	-	-
LAD	1,34	0,27-6,80	0,705	-	-	-

IC: intervalo de confiança; EIM: espessura da íntima média; DAE: diâmetro do átrio esquerdo; EPPVE: espessura da parede posterior do ventrículo esquerdo; ESVE: espessura do septo ventricular esquerdo; VOP: velocidade de onda de pulso; OR: odds ratio; * p < 0,05.

A VOP foi significativamente maior em indivíduos com HVE, com EIM mais elevada, e indivíduos com placa de carótida, estenose igual ou maior que 50%, e com LOA ( Tabela 5 ).

**Tabela 5:** – Comparação da velocidade de onda de pulso de acordo com variáveis de Doppler de carótida e presença ou não de lesões de órgãos-alvo

Variável	Grupo	n	VOP	IC	p
**HVE** ^†^	Não	212	7,6	7,55 - 8,03	<0,0001*
	Sim	105	9,1	8,74 – 9,53	
**EIM** ^‡^	≤ 1 mm	152	8,07	7,79 - 8,35	0,006
	> 1 mm	26	9,12	8,32 - 9,90	
**Presença de placa** ^‡^	Não	82	7,44	7,14 - 7,75	<0,0001*
	Sim	172	9,09	8,83 - 9,35	
**Tamanho da placa** ^‡^	< 50%	146	8,92	8,64 - 9,20	0,003
	≥ 50%	25	10,0	9,42 - 10,63	
Lesões de órgãos-alvo** ^,‡^	Não	118	6,9	6,62 - 7,12	<0,0001*
	Sim	237	8,9	8,69 - 9,17	

IC: intervalo de confiança; EIM: espessura da íntima média; HVE: hipertrofia do ventrículo esquerdo; VOP: velocidade de onda de pulso; *p < 0,05. ^† ^Teste de Mann-Whitney. ^‡ ^Teste t para amostras independentes. ** EIM>1mm, presença de placa, HVE ou VOP > 10 m/s.

## Discussão

No presente estudo, a VOP correlacionou-se com todos os biomarcadores avaliados, e se associou com EIM, mesmo após ajuste para idade e pressão sistólica periférica. A chance de apresentar VOP acima de 10 m/s foi 3,94 vezes maior na presença de EIM maior que 1mm. A VOP apresentou um aumento linear com a presença e tamanho da placa de ateroma, e com a presença de LOA. Esses resultados estão de acordo com os de estudos publicados anteriormente,^2 , 20 , 21^ e reforçam o valor desse biomarcador e sua capacidade de identificar precocemente lesões cardiovasculares, além de seu excelente custo-benefício.

A correlação da VOP com parâmetros ecocardiográficos encontrada no presente estudo pode ser explicada pelo fato de que a rigidez arterial aumenta a PAS, o que causa um retorno precoce das ondas de pulso na sístole em vez de na diástole, e aumento da pós-carga do ventrículo esquerdo. Esse aumento de carga imposto sobre o miocárdio promove hipertrofia cardíaca, e consequente hipertrofia ventricular.^22 - 24^

A HVE, a qual pode ser identificada pelo aumento na espessura da parede do ventrículo esquerdo no ecocardiograma, correlaciona-se com a VOP, e valores de VOP são significativamente maiores em indivíduos com HVE.^22 , 23^ A sobrecarga sobre o ventrículo esquerdo é uma das principais causas de eventos cardiovasculares relacionados com a PAC.^25^

Muitos estudos apresentam não só uma correlação^26 - 28^ como também uma associação entre rigidez arterial e HVE.^22 , 23 , 29 - 32^ Portanto, a rigidez arterial aumentada pode ser usada como preditor de HVE, contribuindo para a prevenção e diagnóstico dessa condição.^23^

Em nosso estudo, não observamos uma associação independente da VOP com ESVE, EPPVE ou DAE, possivelmente por não termos realizado uma análise de associação entre hipertrofia e VOP como nos estudos citados, mas sim entre parâmetros ecocardiográficos e VOP. Ainda, em um dos estudos citados,^22^ foram utilizados achados eletrocardiográficos e não resultados ecocardiográficos, e a maioria dos estudos realizou essa análise de associação com base no índice da massa ventricular esquerda.^23 , 28 , 30 , 32^

A relação entre o aumento da rigidez arterial e o aumento da EIM pode ser explicada pela fisiopatologia da rigidez arterial, que engloba mudanças na matriz extracelular da camada média (túnica média), incluindo quebra de elastina, depósito de colágeno, e reticulação.^24 , 33^ Tais alterações morfológicas também estão relacionadas com envelhecimento vascular.^34^

Um aumento da EIM também está associado com a presença de fatores de risco para arteriosclerose; e idade, pressão arterial, lipídios séricos, e níveis de glicemia de jejum são todos preditores independentes de aterosclerose de carótida.^35^ EIM aumentada é uma das manifestações subclínicas da arteriosclerose.^36^ Existe uma associação independente entre a presença de múltiplos fatores de risco cardiovasculares com aumento na EIM e redução da complacência arterial.^37^

A correlação^38^ e a associação da EIM^38 , 39^ com a VOP também foram previamente relatadas na população idosa.

A avaliação da EIM e da VOP pode aumentar a reclassificação de risco, e esses biomarcadores podem ser utilizados na identificação de LOA.^40^ A combinação desses biomarcadores aumenta o poder preditivo de eventos cardiovasculares em idosos, fornecendo novas informações clínicas importantes.^41^

Em nosso estudo, valores significativamente maiores de VOP foram identificados em indivíduos com estenose igual ou maior que 50%. Valores mais elevados da VOP também se associaram significativamente com presença de placas de carótida.^36^ Além disso, uma redução na elasticidade da carótida está associada com presença de placas e risco de acidente vascular cerebral.^42^

A avaliação combinada de EIM e presença de placas melhora a predição de risco cardiovascular, e a avaliação quantitativa de placas aumenta ainda mais a sensibilidade preditiva.^43^Ainda, a VOP na carótida femoral e o número de placas de ateroma estão associados de maneira significativa e independente com morte cardiovascular, e pode melhorar a identificação de indivíduos em alto risco cardiovascular.^44^

Além das associações entre VOP e biomarcadores, a diferença significativa na VOP encontrada entre indivíduos com e sem LOA destaca a capacidade da VOP em detectar a lesão precocemente. A rigidez arterial é um preditor independente de mortalidade tanto para diabéticos como para a população em geral, e está relacionada com desenvolvimento e progressão de LOA.^45^

A rigidez arterial, avaliada pela VOP, associa-se independentemente com a presença de LOA subclínica, incluindo calcificação da artéria coronária, índice tornozelo-braquial reduzido (doença arterial periférica), e hiperintensidade da substância branca (doença arterial cerebral).^46^

Quando a LOA está presente, mas não é identificada, muitos pacientes são erroneamente classificados como em risco baixo a médio, quando na verdade estão em um risco cardiovascular alto.^47^

As ferramentas diagnósticas devem ser aprimoradas e estabelecidas para a identificação precoce de um risco aumentado, para prevenir o início de LOA e suas complicações. A identificação apropriada de indivíduos com baixo risco é igualmente importante para evitar tratamentos desnecessários e seus efeitos colaterais.^48^ O uso de biomarcadores vasculares é um método custo-efetivo, com valor agregado, na melhoria da identificação de indivíduos em alto risco, facilitando, assim, a prevenção de DCV.^44^

As limitações deste estudo incluem: (1) quando o diagnóstico de diabetes mellitus, dislipidemia e hipertensão arterial não estava disponível nos prontuários médicos, o diagnóstico foi feito durante o estudo, de maneira *ad hoc* , o que pode ter subestimado ou superestimado as frequências dessas doenças. (2) Algumas variáveis de exposição também estavam ausentes nos prontuários médicos. (3) Ainda, não podemos assegurar que todos os pacientes foram submetidos ao Doppler e à ecocardiografia no mesmo local e com o mesmo avaliador. Hipertrofia não pode ser detectada pelo índice da massa ventricular, uma vez que essa informação também não estava disponível nos prontuários.

Os prontuários médicos e os critérios diagnósticos foram avaliados com rigor científico, e os dados foram revisados por dois pesquisadores, com verificação cruzada. Todos esses procedimentos devem validar nossos achados.

O presente estudo destaca a importância do uso da VOP para a detecção precoce de rigidez arterial e LOA, com foco no aumento da EIM, presença de placas de carótida, e HVE. Em geral, a VOP pode otimizar a estratificação do risco cardiovascular para facilitar a intervenção precoce e prevenir DCV e suas complicações.

## Conclusões

A VOP correlacionou-se significativamente com a EIM e com parâmetros ecocardiográficos, e se associou com a EIM. Uma EIM maior que 1mm aumentou a chance de se apresentar uma VOP maior que 10m/s em 3,94 vezes. A VOP foi maior nos indivíduos com HVE, EIM maior que 1mm, com estenose igual ou maior que 50%, e pacientes com LOA.
