# Supplementary material for: Relação entre Velocidade de Onda de Pulso e Biomarcadores Cardiovasculares em Pacientes com Fatores de Risco
Source: Arq Bras Cardiol. 2020 Dec 1;115(6):1125–32. [Article in Portuguese] doi: 10.36660/abc.20190348 (PMC8133733; doi:10.36660/abc.20190348)
Supplement: Translation

# Translation: Relationship between Pulse Wave Velocity and Cardiovascular Biomarkers in Patients with Risk Factors

**Authors:** Rayne Ramos Fagundes, Priscila Valverde Oliveira Vitorino, Ellen de Souza Lelis, Paulo Cesar B. Veiga Jardim, Ana Luiza Lima Souza, Thiago de Souza Veiga Jardim, Pedro Miguel Guimarães Marques Cunha, Weimar Kunz Sebba Barroso

**Affiliations:** 1 Brazil Universidade Federal de Goiás, Goiânia, GO – Brazil; 2 Brazil Pontifícia Universidade Católica de Goiás - Escola de Ciências Sociais e da Saúde, Goiânia, GO - Brazil; 3 Brazil Universidade Federal de Goiás - Liga de Hipertensão, Goiânia, GO - Brazil; 4 Portugal Universidade do Minho Escola de Medicina, Braga – Portugal

**Keywords:** Cardiovasclar Diseases/mortality, Blood Pressure, Risk Factors, Hypertension, Left Ventricle Dysfunction, Diabetes Mellitus

## Abstract

**Background:**

The relationship between pulse wave velocity (PWV) and biomarkers of structural changes of the left ventricle and carotid arteries remains poorly understood.

**Objective:**

To investigate the relationship between PWV and these biomarkers.

**Methods:**

This was an analytical, retrospective, cross-sectional study. Medical records of patients with diabetes mellitus, dyslipidemia, and pre-hypertension or hypertension, who underwent central blood pressure (CBP) measurement using Mobil-O-Graph®, and carotid doppler or echocardiography three months before and after the CBPM were analyzed. Statistical analysis was performed using Pearson or Spearman correlation, linear bivariate and multiple regression analysis, and the t test (independent) or Mann-Whitney test. A p <0.05 indicated statistical significance.

**Results:**

Medical records of 355 patients were analyzed, mean age 56.1 (±14.8) years, 51% male. PWV was correlated with intima-media thickness (IMT) of carotids (r=0.310) and left ventricular septal thickness (r=0.191), left ventricular posterior wall thickness (r=0.215), and left atrial diameter (r=0.181). IMT was associated with PWV adjusted by age and peripheral systolic pressure (p=0.0004); IMT greater than 1 mm increased the chance of having PWV above 10 m/s by 3.94 times. PWV was significantly higher in individuals with left ventricular hypertrophy (p=0.0001), IMT > 1 mm (p=0.006), carotid plaque (p=0.0001), stenosis ≥ 50% (p=0.003), and target-organ damage (p=0.0001).

**Conclusion:**

PWV was correlated with IMT and echocardiographic parameters, and independently associated with IMT. This association was stronger in individuals with left ventricular hypertrophy, increased IMT, carotid plaque, stenosis ≥ 50%, and target organ damage. (Arq Bras Cardiol. 2020; 115(6):1125-1132)

## Introduction

The high prevalence and mortality of cardiovascular diseases (CVD) highlights the urgent need to implement tools to better stratify cardiovascular risks, to identify patients at high risk, and to diagnose and treat CVD in early stages. One of these tools are cardiovascular biomarkers, which can detect CVD in a subclinical phase with good accuracy, thereby improving the prevention of events and the epidemiological scenario.^1 , 2^

Some of the main biomarkers related to vascular structure and function are intima-media thickness (IMT), the presence of carotid plaques, pulse wave velocity (PWV), and the ankle-brachial index (ABI).^2^In addition, other cardiovascular biomarkers are used to identify target-organ damage (TOD), such as left ventricular hypertrophy, elevated serum creatinine levels, increased albumin excretion, and reduced glomerular filtration rate.^3 , 4^

PWV, a vascular damage biomarker used to assess arterial stiffness, is considered a strong and independent marker of TOD and adverse events.^5^PWV is also a predictor of all-cause mortality, indicating the patient’s actual risk.^6^Each one meter per second rise in PWV leads to an increases by 14% in the risk of adverse events and by 15% in the cardiovascular risk and all-cause mortality.^6^Among its advantages, PWV is non-invasive, easily performed, relatively inexpensive, and widely validated method^2^with clearly established reference values.^7 , 8^Despite this evidence, PWV remains underused in clinical practice, and few studies have analyzed its relationship with other biomarkers, specially using oscilometric method. Thus, the objective of this study was to investigate the relationship between PWV and other biomarkers of cardiovascular structural changes in patients with cardiovascular risk factors.

## Methods

### Participants

From September 2012 to March 2017, 660 central blood pressure (CBP) measurements were performed. Among these evaluations, 131 patients performed the examination two times or more, for a total of 169 repeated evaluations. Therefore, the study population consisted of 491 patients that underwent CBP measurement to restratify patients considered as low or intermediate cardiovascular risk.

The sample was calculated considering a 5% error and a 95% confidence level, indicating a minimum sample of 216 patients. Finally, the study sample consisted of 355 Brazilian patients referred to cardiology clinic for CBP measurements ( Figure 1 ).

**Figure 1:** – Study sample selection flowchart. CBPM, central blood pressure measurement; DM: diabetes mellitus; DLP: dyslipidemia; PH: pre-hypertension; HT: hypertension.

### Study Design and Procedures

This analytical, retrospective, cross-sectional study was performed by analysis of medical records and test reports. Data were first collected from the medical records contained in the institutional archives. The following exclusion criteria were applied: age younger than 18 years; absence of the following diagnoses: diabetes mellitus (DM), dyslipidemia (DLP), pre-hypertension (PH) or hypertension (HT); absence of a carotid Doppler or an echocardiography in the three months before and after CBP measurement ( Figure 1 ).

Then, the diagnoses of all patients were retrieved from the medical records; when the diagnoses were not available, the diagnostic criteria were used – fasting blood glucose levels > 125 mg/dL or use of hypoglycemic drugs for DM; triglyceride levels > 150 mg/dL and low-density lipoprotein (LDL) > 100 mg/dL and/or high-density lipoprotein (HDL) < 40 mg/dL and/or current use of statins were considered dyslipidemic. Individuals with peripheral systolic blood pressure (SBP) ranging from 121 to 139 mmHg and DBP ranging from 81 to 89 mmHg, obtained during CPB measurement procedures, were classified as pre-hypertensive and those with blood pressure equal to or higher than 140/90 mmHg were classified as hypertensive.^4^

Data on the following other variables were collected from the medical records: sex (female or male), tobacco smoking (yes or no) and marital status (with partner or without partner). In addition to the results of imaging tests, results of carotid Doppler and/or echocardiogram studies conducted in the three months before or after the CBP examination were analyzed. When those studies were performed more than once in this period, results of the last test before the CBP measurement was considered for analysis.

### Central Blood Pressure Measurement

CBP was measured non-invasively using the validated oscilometric Mobil-O-Graph NG (IEM, Stolberg, Germany) with inbuilt ARCSolver algorithm.^10^All the CBPM procedures were performed by the same person, always between 1 p.m and 2 p.m. The measurements were made using triple pulse wave analysis and calibration MAD-c2 (mean arterial diastolic blood pressure).^9 , 10^

Chronological age was calculated as the difference between the date of birth and the date of the CBP measurement. Weight (kg) and height (m) were used for body mass index calculation (using Quetelet formula)^11^and its subsequent classification.^12^Peripheral SBP (SBPp), peripheral DBP (DBPp), central SBP (SBPc), augmentation index (AIx), and PWV were also analyzed.^13^All patients were instructed not to smoke or drink coffee before the test.

### Carotid Doppler and Echocardiogram

Imaging examinations were performed at different imaging centers, determined by patient’s choice. Those performed at the cardiology clinic where data collection was performed, were conducted using the Philips HD 11 ultrasound machine.

The carotid Doppler was performed following the American^14^and European^15^consensus guidelines, and the highest values obtained from the left and right common carotid arteries were considered for statistical analysis purposes.

Echocardiographic parameters were assessed by two-dimensional transthoracic echocardiography,^16^measuring the left ventricular septal thickness (LVST), the left ventricular posterior wall thickness (LVPWT), and the left atrial diameter (LAD).

### Target-organ Damage

The identification of TOD was based on the presence of increased IMT,^17^atheroma plaques in the carotid Doppler,^3 , 4^left ventricular hypertrophy (LVH) in the echocardiogram,^18^and increased arterial stiffness identified by a PWV higher than 10 m/s^3 , 4^( Figure 2 ).

**Figure 2:** – Examinations and reference values considered indicative of target organ damage. LAD: left atrial diameter; IMT: intima-media thickness; LVPWT: left ventricular posterior wall thickness; LVST: left ventricular septal thickness; PWV: pulse wave velocity.

### Statistical Analysis

Data were collected and scanned in duplicate by two researchers, using Epidata software, version 3.1. After assessing and correcting inconsistencies, the data were exported to the Statistical Package for Social Science (SPSS), version 18.0. The Kolmogorov-Smirnov test was applied, and a descriptive data analysis was performed. Statistical analysis was performed based on data distribution, using parametric and non-parametrical tests. Numeric data were described as mean and standard deviation or median and interquartile range, depending on data distribution. Categorical variables were presented with absolute and relative frequencies. The Pearson product-moment correlation or Spearman’s rank-order correlation were used to assess the correlation of PWV with the results of the carotid Doppler and the echocardiogram. Correlations were classified in weak (0 < r <0.30), moderate (0.30 ≤ r < 0.60), strong (0.60 ≤ r < 0.90) and very strong (0.90 ≤ r < 1).^19^

The association between PWV and the other biomarkers (IMT, LVST, LVPWT, LAD) was assessed by linear bivariate regression analysis and those variables with p<0,020 were used in multiple regression analysis. All assumptions were met for the application of linear regression analysis. PWV was compared by IMT size, with the presence or not of LVH, with the presence or not of plaque, with plaque size, and with the presence or not of TOD using the *t* test for independent samples or Mann-Whitney test. Values of p<0.05 were considered statistically significant.

### Ethical Aspects

The study was conducted in accordance with the 466/12 resolution of the Brazilian National Council of Health and was approved by the Ethics Committee of the Hospital das Clínicas da Universidade Federal de Goiás (UFG), under approval number 1.500.463.

## Results

In total, 355 individuals with a mean age of 56.1 (±14.8) years participated in this study. Most of them had dyslipidemia and/or arterial hypertension, 148 (41.7%) were overweight and 130 (36.6%) were obese ( Table 1 ).

**Table 1:** – Sample characterization (n=355)

Variables		Mean (SD) / Median (25%-75%) / n (%)
Age		56.1 (±14.8)
BMI		28.7 (±4.9)
SBPc		113 (107-123)	
Alx		21.5 (±13.4)	
PWV		8.2 (±2)	
Sex				
Male		181 (51%)
Female		174 (49%)
**Marital status**		
With partner		251 (70.7%)
Without partner		102 (28.7%)
**CVRF**		
Overweight		148 (41.7%)
Obesity		130 (36.6%)
Tobacco smoking		12 (3.4%)
**Diagnosis**		
Dyslipidemia		306 (86.2%)
Arterial hypertension		283 (79.7%)
Diabetes mellitus		65 (18.3%)
Pre-hypertension		47 (13.2%)

*Aix: augmentation index; BMI: body mass index; CVRF: cardiovascular risk factors; PWV: pulse wave velocity; SBPc: central systolic blood pressure.*

A moderate and positive correlation was found between PWV and IMT; and positive and weak correlations were identified between PWV and LVST, and between LVPWT and LAD ( Table 2 ).

**Table 2:** – Correlation of pulse wave velocity with cardiovascular biomarkers

		IMT (n=178)	LVST (n=313)	LVPWT (n=312)	LAD (n=312)
PWV	r	0.310^†^	0.191^†^	0.215^†^	0.181^‡^
	p	<0.001*	0.001*	<0.001*	0.001*

*p < 0.05.^†^Spearman’s rank-order correlation;^‡^Pearson product-moment correlation. IMT: intima-media thickness; LAD: left atrial diameter; LVPWT: left ventricular posterior wall thickness; LVST: left ventricular septal thickness; PWV: pulse wave velocity.

IMT was associated with PWV adjusted by age and peripheral systolic pressure (p=0.0004), such that IMT greater than 1 mm increased by 3.94 the chance of having PWV above 10 m/s ( Tables 3 and 4 ).

**Table 3:** – Linear bivariate regression analysis of pulse wave velocity with the cardiovascular biomarkers

Variables	OR	95%CI (OR)	p
LVST	2.49	1.38 – 4.49	0.003*
IMT	3.94	1.53 – 10.15	0.004*
LVPWT	2.34	1.29 – 4.22	0.005*
LAD	2.55	1.18 – 5.49	0.017*

Linear bivariate regression analysis. CI: confidence interval; IMT: intima-media thickness; LAD: left atrial diameter; LVPWT: left ventricular posterior wall thickness; LVST: left ventricular septal thickness; OR, odds ratio; * p < 0.05.

**Table 4:** – Multiple regression analysis of pulse wave velocity with the cardiovascular biomarkers

Variables	Adjusted OR	95%CI (OR)	p	Adjusted OR*	95%CI (OR)	p
LVST	1.64	0.59-4.5	0.340	-	-	-
IMT	3.94	1.53- 10.15	0.004	6.86	1.78-26.45	<0.001
LVPWT	1.69	0.64-4.49	0.294	-	-	-
LAD	1.34	0.27-6.80	0.705	-	-	-

Multiple regression analysis. CI: confidence interval; IMT: intima-media thickness; LAD: left atrial diameter; LVPWT: left ventricular posterior wall thickness; LVST: left ventricular septal thickness; OR, odds ratio; * p < 0.05.

PWV was significantly higher in individuals with LVH, higher IMT, in those with carotid plaque, in those with stenosis equal to or greater than 50%, and in those with TOD ( Table 5 ).

**Table 5:** – Comparison of pulse wave velocity according to carotid Doppler variables and presence or not of target organ damage

Variable	Group	n	PWV	CI	p
**LVH** ^†^	No	212	7.6	7.55 - 8.03	<0.0001*
	Yes	105	9.1	8.74 – 9.53	
**IMT** ^‡^	≤ 1 mm	152	8.07	7.79 - 8.35	0.006
	> 1 mm	26	9.12	8.32 - 9.90	
**Presence of plaque** ^‡^	No	82	7.44	7.14 - 7.75	<0.0001*
	Yes	172	9.09	8.83 - 9.35	
**Plaque size** ^‡^	< 50%	146	8.92	8.64 - 9.20	0.003
	≥ 50%	25	10.0	9.42 - 10.63	
Target organ damage** ^,‡^	No	118	6.9	6.62 - 7.12	<0.0001*
	Yes	237	8.9	8.69 - 9.17	

CI: confidence interval; IMT: intima-media thickness; LVH: left ventricular hypertrophy; PWV: pulse wave velocity. *p < 0.05.^†^Mann-Whitney test.^‡^t-test for independent samples. ** IMT>1mm, presence of plaque, LVH or PWV > 10 m/s.

## Discussion

In the present study, PWV was correlated with all biomarkers evaluated, and associated with IMT even when adjusted for age and peripheral systolic pressure. The chance of having PWV above 10 m/s increases by 3.94 times in the presence of IMT greater than 1 mm. PWV have had a linear increment with the presence and size of atheroma plaque and with the presence of TOD. These findings are in accordance with previously published studies,^2 , 20 , 21^and reinforce the value of this biomarker and its ability to identify early cardiovascular damage, in addition to its excellent cost-effectiveness.

The correlation of PWV with echocardiographic parameters observed in the present study may be explained by the fact that arterial stiffness increases the SBP, and, consequently, the reflected wave returns early and arrives in systole instead of diastole, increasing the post-load of the left ventricle. This increased workload imposed on the myocardium promotes cardiac myocyte hypertrophy, resulting in ventricular hypertrophy.^22 - 24^

LVH, which may be identified by an increase in left ventricular wall thickness on echocardiogram, is correlated with PWV, and PWV values are significantly higher in individuals with LVH.^22 , 23^The increase in the load imposed on the left ventricle is one of the main causes of cardiovascular events related to CBP.^25^

Many studies show not only a correlation^26 - 28^but also an association between arterial stiffness and LVH.^22 , 23 , 29 - 32^Therefore, increased arterial stiffness may be used as a predictor of LVH, contributing to the prevention and diagnosis of this condition.^23^

In our study, PWV was not independently associated with LVST, LVPWT, or LAD, perhaps because the association analysis was not performed between hypertrophy itself and PWV, as in the studies cited, but rather between echocardiographic parameters and PWV. Furthermore, one of the studies cited^22^used electrocardiographic and not echocardiographic findings, and most studies performed this association analysis based on the left ventricular mass index.^23 , 28 , 30 , 32^

The relationship between the increases in arterial stiffness and the increases in IMT can be explained by the pathophysiology of arterial stiffness, which encompasses changes in the extracellular matrix of the middle layer (tunica media), including elastin breakdown, collagen deposition, and reticulation.^24 , 33^Those morphological changes are also related to vascular aging.^34^

Increased IMT is also associated with the presence of risk factors for arteriosclerosis; and age, arterial blood pressure, serum lipids, and fasting blood glucose levels are all independent predictors of carotid atherosclerosis.^35^Increased IMT is one of the first subclinical manifestations of arteriosclerosis.^36^The presence of multiple cardiovascular risk factors is independently associated with increases in IMT and decreases in arterial compliance.^37^

The correlation^38^and association of IMT with PWV was also previously reported in an elderly population.^38 , 39^

The assessment of IMT and PWV can improve cardiovascular risk reclassification, and these biomarkers may be used to identify subclinical TOD.^40^The combination of these biomarkers increases the predictive power of cardiovascular events among elderly people, providing additional and important clinical information.^41^

Significantly higher PWV values were also identified in individuals with stenosis equal to or greater than 50% in the present study. Higher PWV values were also significantly associated with the presence of carotid plaques.^36^Furthermore, decreased carotid elasticity is associated with the presence of plaques and the risk of stroke.^42^

The combined assessment of IMT and the presence of plaques improves the prediction of cardiovascular risk, and the quantitative evaluation of plaques further increases the predictive sensitivity.^43^Moreover, femoral carotid PWV and the number of atheroma plaques are significantly and independently associated with cardiovascular death and can improve the identification of individuals at high cardiovascular risk.^44^

In addition to the associations between PWV and biomarkers, the significant difference in PWV found between study subjects with and without TOD highlights the capacity of PWV to early identify the damage. Arterial stiffness is an independent predictor of mortality for both diabetics and the general population, and it is related to TOD development and progression.^45^

Arterial stiffness, assessed by PWV, is independently associated with the presence of subclinical TOD, including coronary artery calcification, reduced ankle-brachial index (peripheral arterial disease), and white matter hyperintensity (cerebral arterial disease).^46^

When TOD is present and is not identified, many patients are wrongly classified as medium-to-low risk when they are actually at high cardiovascular risk.^47^

Diagnostic tools must be improved and established for the early identification of increased risk, to prevent the onset of TOD and its complications. The appropriate identification of low-risk individuals is equally as important to avoid unnecessary treatments and their concomitant side effects.^48^The use of vascular biomarkers is a cost-effective, value-added method of improving the identification of individuals at high cardiovascular risk, thereby facilitating the prevention of CVD.^44^

The limitations of this study are as follows: when diagnosis of diabetes mellitus, dyslipidemia, and arterial hypertension was not available in the medical records, the diagnosis was made during the study, on an ad hoc basis, which may have under- or overestimated the frequencies of those diseases. Some exposure variables were missing from the medical records. In addition, we cannot ensure that all patients underwent carotid Doppler and echocardiography at the same clinic and with the same evaluator. Hypertrophy could not be detected by the ventricular mass index, since this information was not available in the medical records.

The medical records and diagnostic criteria were reviewed with scientific rigor, and the data were reviewed by two researchers and subsequently crosschecked. All these procedures should suffice to validate the findings.

The present study highlights the importance of using PWV for the early detection of arterial stiffness and TOD, focusing on increases in IMT, the presence of carotid plaques, and LVH. Overall, the use of PWV may optimize the stratification of cardiovascular risk to facilitate early intervention and prevent CVD and its complications.

## Conclusions

PWV was significantly correlated with IMT and echocardiographic parameters and was associated with IMT. Furthermore, an IMT greater than 1 mm increased the chance of having PWV above 10 m/s by 3.94 times. PWV was significantly higher in individuals with LVH, IMT greater than 1 mm, in those with stenosis equal to or greater than 50%, and in those with TOD.
